# Case Report: Achieving ankle joint stability through early intervention in an 8-year-old with congenital fibular hemimelia

**DOI:** 10.3389/fped.2025.1528401

**Published:** 2025-05-30

**Authors:** Yanhong Ma, Na Pan, Gang Peng, Rui Yang

**Affiliations:** ^1^ Department of Pediatric Orthopedics, Shanghai Children’s Medical Center GuiZhou Hospital, Shanghai Jiao Tong University School of Medicine, Guiyang, China; ^2^ Department of Pediatric Surgery, Guizhou Provincial People’s Hospital, Guiyang, China

**Keywords:** congenital fibular hemimelia, the stability of ankle joint, conservative treatment, child, case report

## Abstract

**Introduction:**

Congenital Fibular Hemimelia (CFH), also known as congenital absence of the fibula, has an incidence of approximately 5.7–20 cases per million live births. Clinically, it manifests as partial or complete absence of the fibula, accompanied by tibial shortening and bowing, ball-and-socket or dish-shaped ankle joints, and tarsal anomalies. Surgical intervention serves as the primary therapeutic approach for CFH. Most pediatric patients require at least two surgical procedures, with the initial stage involving ankle reconstruction surgery to achieve joint stability, followed by subsequent limb lengthening procedures to correct limb length discrepancies between the lower extremities.

**Presentation of case:**

Here, we report for the first time a case of CFH with complete absence of fibula in a pediatric patient who achieved ankle stability through early application of splinting to maintain the right ankle in a functional position, thereby correcting valgus deformity and obviating the need for ankle reconstruction surgery. Following admission, the patient underwent limb lengthening surgery exclusive Ilizarov-based limb lengthening, achieving favorable clinical outcomes during early-to-mid postoperative follow-up.

**Discussion:**

Compared with the conventional “two-stage” surgical protocol for CFH, early conservative management demonstrates efficacy in correcting foot valgus deformity, thereby circumventing the need for multiple surgical interventions and associated morbidities in pediatric patients.

**Conclusion:**

This case report suggests that early conservative treatment may correct ankle instability in patients with CFH, thereby preventing the necessity for multiple complex surgical procedures. These findings highlight the critical importance of early screening and intervention, while providing novel insights into therapeutic paradigms for CFH management.

## 1 Introduction

Congenital fibular hemimelia (CFH) is a common congenital limb deficiency but still classified as a rare disease, with an incidence of approximately 1:135,000 to 1:5,000 in live births. Clinically, it manifests as partial or complete absence of the fibula, accompanied by shortening and bowing of the tibia, ball-and-socket or dish-shaped ankle joint, and tarsal anomalies (1). During growth and development, patients often exhibit progressive valgus deformity of the foot and lower limb length discrepancy, which severely impacts their quality of life (2). The main treatment goals for CFH patients are limb length equalization, correction of foot and ankle deformities, and gait improvement.

The pathological basis of the series of deformities caused by CFH is the replacement of the fibula by a fibrous cartilaginous band. Due to the tethering effect of this band, tibial development is delayed, leading to anteromedial bowing of the tibia. The absence of the lateral malleolus and the contracture and tethering of the peroneal muscles and Achilles tendon result in valgus deformity of the foot (1, 3). Therefore, early surgical removal of the fibular fibrous band and release of contracted Achilles tendon and peroneal tendons can effectively prevent further aggravation of tibial and foot and ankle deformities in CFH patients. Achieving ankle joint stability through foot and ankle reconstructive surgery is often the first step in the surgical treatment of CFH.

Despite continuous advancements in surgical techniques for CFH (4, 5), patients who originally required amputation or prosthesis can now avoid amputation and achieve better function. However, these surgical methods inevitably require initial soft tissue release or ankle joint reconstruction to obtain a stable ankle joint (6). Consequently, some CFH patients need to undergo multiple surgeries, imposing significant pain and burden on the patients and their families.

Herein, we report an 8-year-old girl with CFH characterized by complete absence of the fibula. Who achieved a stable ankle after early corrective treatment, allowing the patient to undergo only limb length discrepancy correction without soft tissue release or reconstructive surgery for ankle. This approach not only significantly reduced the complexity of the surgery but also minimized the trauma and burden on the patient. To our knowledge, this is the first reported case of CFH in which a stable ankle was achieved through early conservative treatment, thereby avoiding ankle reconstruction surgery. We believe that the early intervention combined with surgical treatment in this case will provide new insights into CFH treatment strategies.

## 2 Case description

The 8-year-and-6-month-old child, born in the economically underdeveloped southwestern region of China, is of Han ethnicity and has no family history of genetic disorders. After birth, the parents found that the child's right foot was obviously valgus, so the parents used a splint to fix the child's right ankle joint in a functional position at home for 3 months. Subsequently, the splint was removed during the day to mobilize the ankle joint, and the splint was used to fix the ankle joint at night. After 6 months of this treatment, the right foot of the patient exhibited a normal appearance. By the age of 18 months, the child demonstrated normal ambulation without observable gait abnormalities. However, as the child grew and developed, the leg length discrepancy became increasingly apparent, resulting in a limping gait, and the patient was subsequently admitted via outpatient consultation.

### 2.1 Physical examination

**Figure 1:**
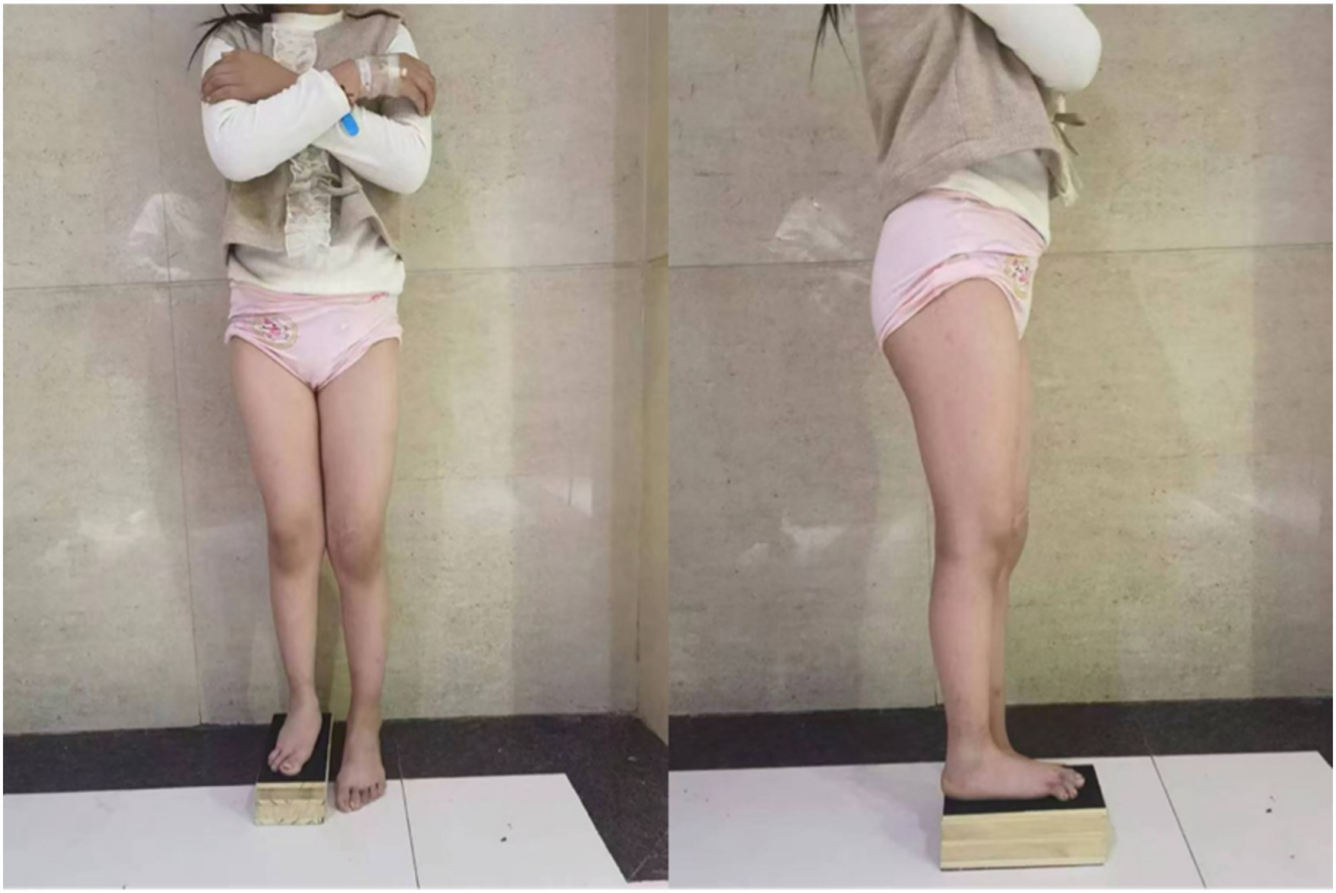
Preoperative clinical photograph of the patient.

The patient walked with a limp, with obvious deformity and shortening of the right calf (Figure 1), without varus or valgus of the right foot, without varus or valgus of the right ankle, without the fourth and fifth phalanx of the right foot, without the right arch of the right foot, without the fibula and lateral malleolus on palpation, and with good mobility of the right hip joint and knee joint. Muscle strength in the right lower limb was grade 5(According to the scale proposed by the Medical Research Council, MRC scale) (7), with normal muscle tone(According to the modified Ashworth scale) (8). Circumferential measurements were as follows: right thigh 38.5 cm, right knee 29 cm, right calf 23 cm, right ankle 15.5 cm; left thigh 39.5 cm, left knee 31 cm, left calf 36 cm, left ankle 19 cm. No abnormalities were found in the left lower limb examination. Negative bilateral anterior drawer tests and normal single-leg stance test results were observed. The patient exhibited a limping gait with a stride length of 40 cm and preserved functional ambulation capability classified as Functional Ambulation Category (FAC) Level 5 (9). A video of the patient walking is provided in Supplementary File S1.

### 2.2 Imageological examination

**Figure 2:**
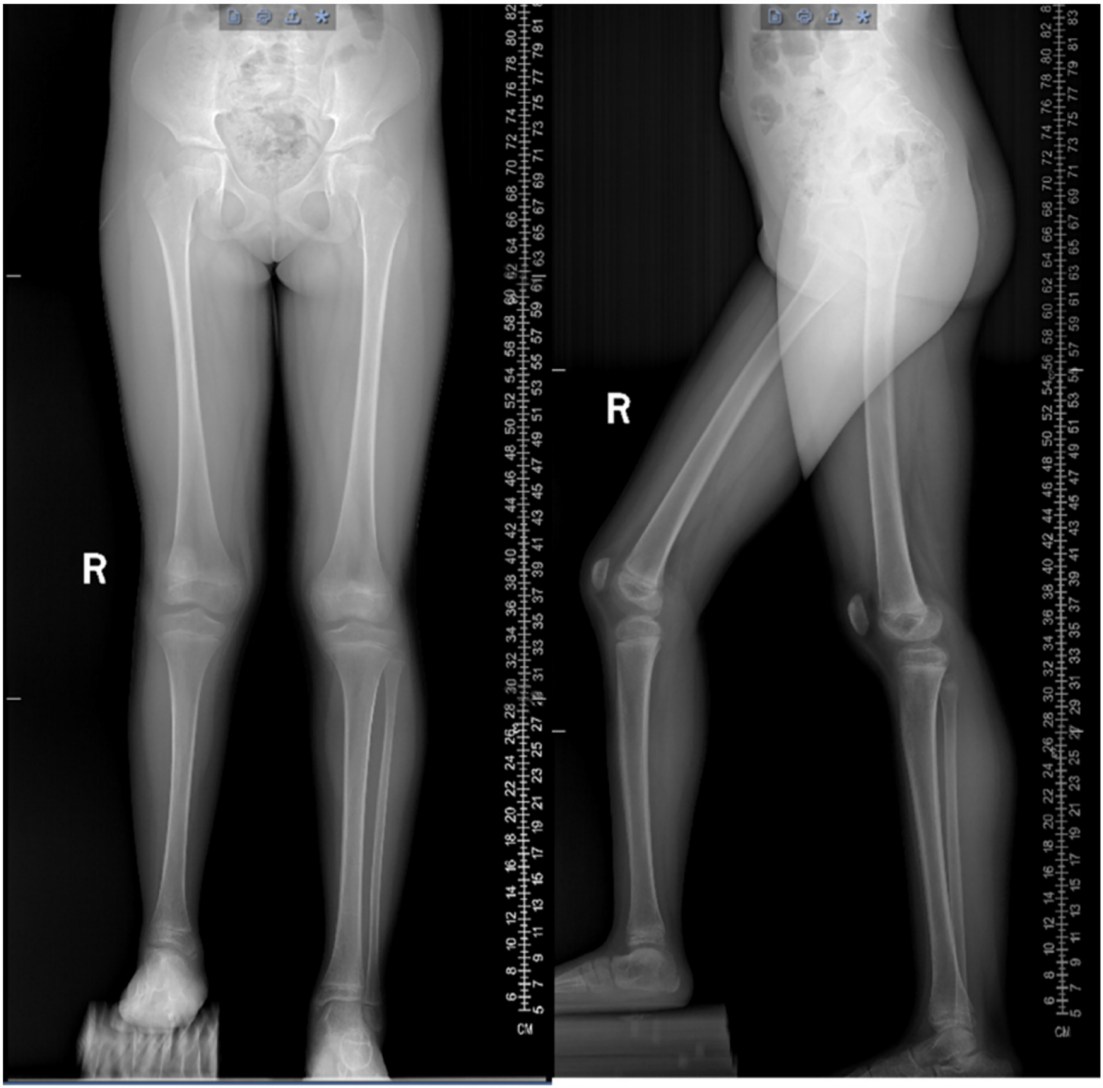
Preoperative anteroposterior and lateral radiographs of both lower limbs. The patient had significant length discrepancy of the lower limbs and complete absence of the right fibula.

**Figure 3:**
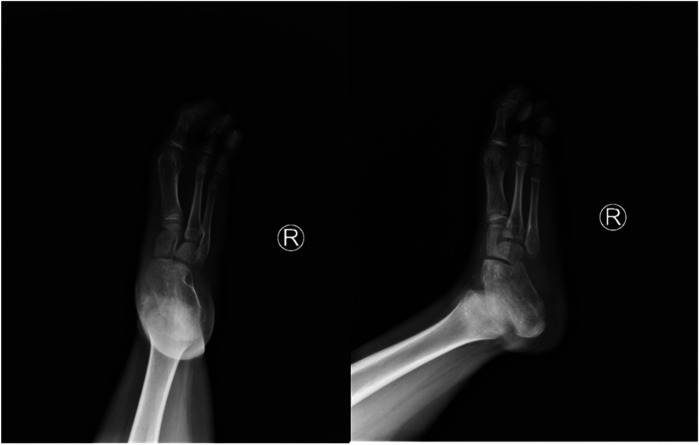
Preoperative anteroposterior and oblique radiographs of the right foot.

**Figure 4:**
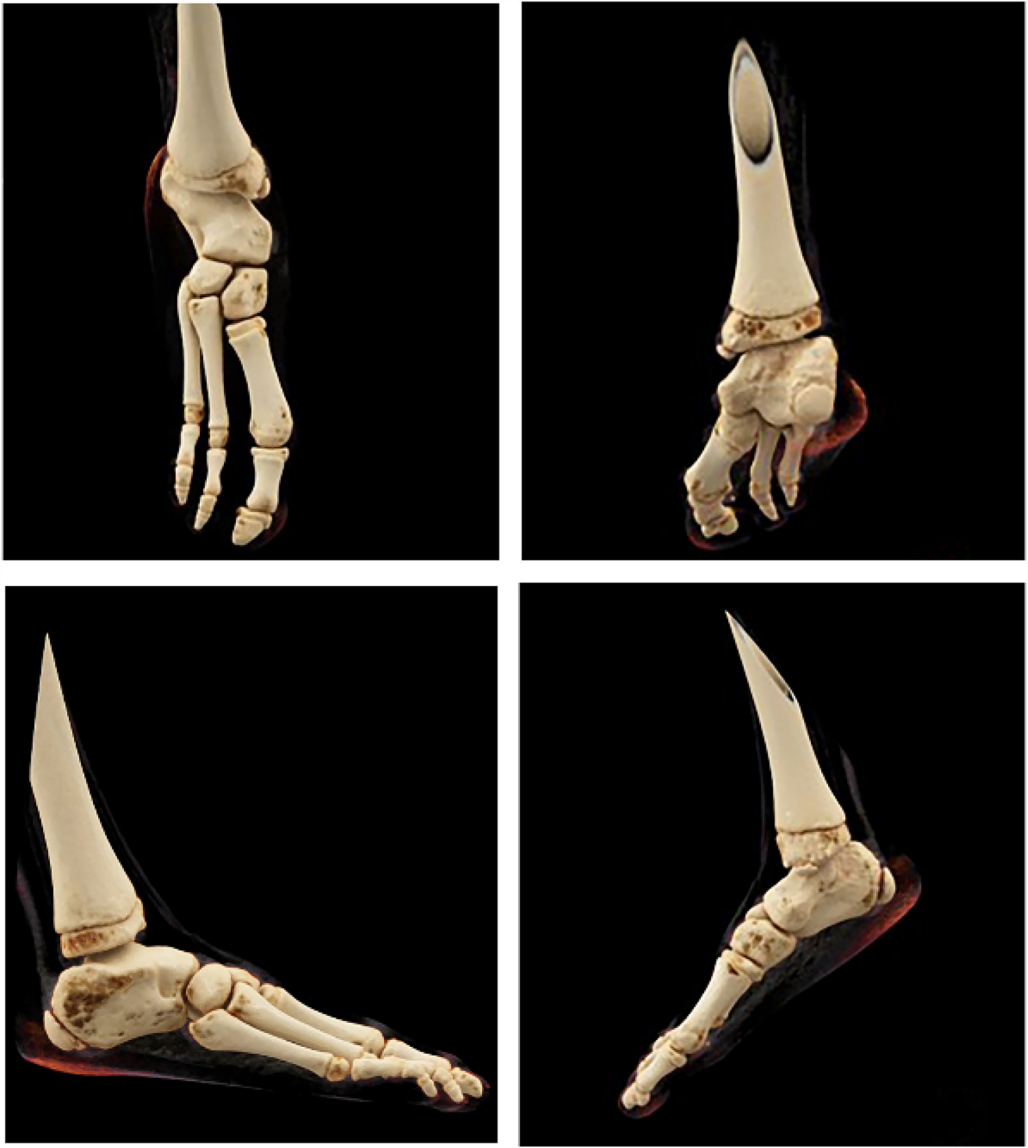
CT of the right foot: absence of the 4th and 5th metatarsals, 4th and 5th phalanges, navicular bone, lateral cuneiform, and cuboid bone; fusion of the right calcaneus and talus.

X-ray (Figures 2, 3) and CT (Figure 4) examinations indicated abnormal morphology of the right lower limb and limb length discrepancy, with the right lower limb approximately 6 cm shorter than the left. The right fibula, the 4th and 5th metatarsals and phalanges, the navicular bone, the lateral cuneiform, and the cuboid bone were absent. Fusion of the right calcaneus and talus was also observed.

### 2.3 Classification and surgery

Based on the medical history, physical examination, and imaging findings, it was considered that the child should be diagnosed with right fibular hemimelia, Paley III type B. Given the stable status of the right ankle joint, ankle reconstruction surgery was deemed unnecessary. The surgical approach adopted was tibial osteotomy and lengthening by Ilizarov technique, involving: (1) Transverse osteotomy at the proximal third of the right tibia; (2) Division of the fibular fibrous band; (3) Application of an Ilizarov circular external fixator.

### 2.4 Follow-up

**Figure 5:**
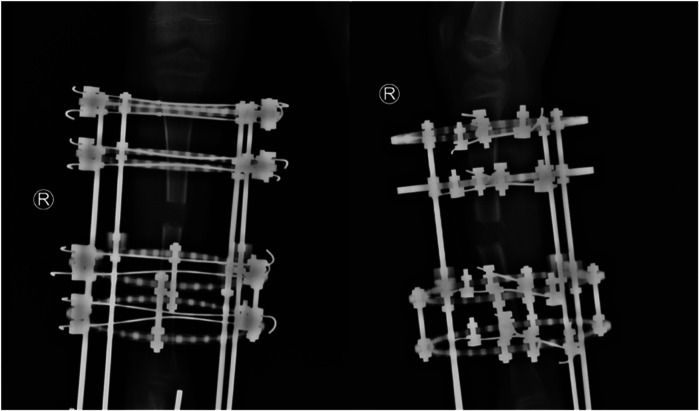
Anteroposterior and lateral radiographs of the right tibia at 1 month postoperatively.

**Figure 6:**
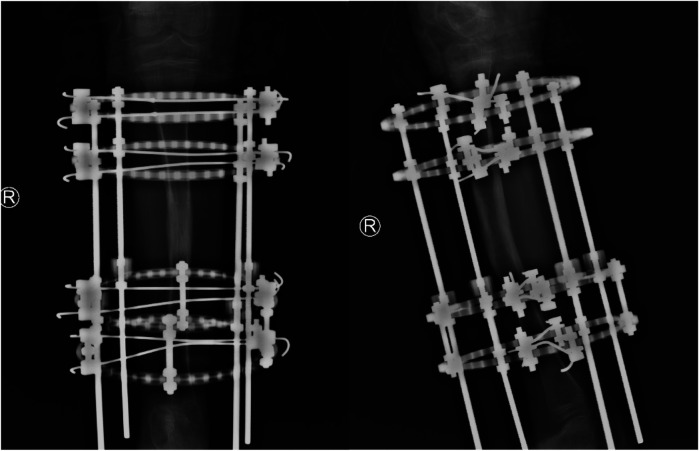
Anteroposterior and lateral radiographs of the right tibia at 6 month postoperatively.

The surgery was successfully performed, with postoperative x-ray evaluations conducted at 10 days, 1 month (Figure 5), and 6 months (Figure 6). At the 6-month follow-up, imaging demonstrated successful bone healing with adequate regenerate formation at the tibial osteotomy site, showing a 5 cm lengthening compared to preoperative measurements. No early-to-mid complications, including pin tract infection, nonunion/poor bone union, or axial deviation, were observed. A planned assessment will be conducted at 9 months postoperatively to determine the necessity of fixator removal. The timeline of key events is as follows (Table 1).

**Table 1:** The timeline of key events: From symptom onset to disease remission.

Date	Event/intervention	Outcome/details
11/2015–02/2016	Found at Birth-Initial Treatment: Severe right foot valgus observed; Parents immediately used a splint to fix the right ankle joint in functional position.	24 h splint immobilization with a treatment duration of three months.
03/ 2016–05/2016	Exercise ankle intermittently: Splint fixation for another 3 months (daytime removal for ankle mobilization, nighttime immobilization).	The right foot exhibited a normal appearance.
05/2017	Normal ambulation	Normal gait achieved by 18 months of age.
25/08/2024	Presentation at Clinic: Progressive limb length discrepancy and limping gait.	Admission for evaluation.
25/08/2024	Diagnostic Workup: x-ray and CT confirmed Congenital Fibular Hemimelia (right limb 6 cm shorter).	Surgical planning initiated.
27/08/2024	Surgery: Tibial osteotomy and lengthening by Ilizarov technique, No ankle reconstruction surgery was required.	No early postoperative complications were observed.
08/09/2024	First Postoperative Check: 10 days after surgery.	Initial radiographic assessment.
09/09/2024	Discharge: Adjusted Ilizarov frame for continued distraction.	Outpatient follow-up schedule established.
26/09/2024	Second Follow-up: x-ray showed about 2.2 cm tibial lengthening at 1 month after surgery.	Adjusted Ilizarov frame for continued distraction.
02/04/2025	Third Follow-up: x-ray confirmed about 5 cm tibial lengthening at 6 month after surgery.	Ongoing monitoring for limb symmetry.
Planned (07/2025)	Fourth Follow-up: Scheduled 9-month post-operation imaging at 9 month after surgery.	Determine the necessity of fixator removal and final limb length assessment.

## 3 Discussion

CFH is the most common congenital longitudinal deficiency of the lower limb, predominantly unilateral, with a higher incidence in males than in females (10). The exact etiology of CFH remains unclear. The widely accepted theory is embryonic vascular dysplasia: during early embryonic development (5–7 weeks of gestation), the limb vasculature begins to develop progressively. The absence of the anterior tibial artery and fibular artery leads to abnormal development of the fibular side of the limb (11). In addition to partial or complete congenital absence of the fibula, CFH often presents with five major deformities (12–14):

(1) Tibial Deformity: Due to congenital tibial dysplasia, the affected tibia usually bows anteromedially during ambulation because of uneven load distribution between the lower limbs.
(2) Genu Valgum: Often caused by hypoplasia of the lateral femoral condyle and lateral tibial plateau, resulting in valgus alignment of the knee joint (15).
(3) Limb Length Discrepancy: This is primarily caused by significant shortening of the affected tibia compared to the contralateral side. Due to the dysplasia of both the femur and tibia on the affected side, limb length discrepancy in CFH patients can exceed 30 cm (10).
(4) Knee Joint Instability: Primarily caused by hypoplasia or absence of the anterior cruciate ligament in the affected limb (16).
(5) Foot and Ankle Deformities: These include ball-and-socket ankle joint, tarsal coalition, and absence of the lateral foot alignment (17, 18).

There are many classification methods for CFH (1). In addition to Stanitski classification, Coventry Johnson classification and Birch classification, Achterman-Kalamchi classification is the most commonly used classification method, which classifies CFH according to the degree of fibula absence. However, the Achterman-Kalamchi classification does not account for associated deformities in CFH patients and offers limited guidance for surgical decision-making (19, 20). Therefore, Paley proposed a new classification based on reconstructive surgical methods (1, 21), dividing CFH into four types: Type I: Stable ankle joint with only mild fibular hypoplasia. Type II: Reducible ankle valgus that can be corrected to a normal position. Type III: Equinovalgus foot deformity, further subdivided into: Type IIIA: Due to malorientation of the ankle joint; Type IIIB: Subtalar joint coalition; Type IIIC: Combination of Types IIIA and IIIB deformities. Type IV: Equinovarus foot deformity.

In the past, the treatment of CFH was mainly amputation and postoperative artificial limb. However, with the development of pediatric orthopedic surgery, the emergence of surgical techniques such as tibial centralization, soft tissue release, and limb lengthening has provided great help for the treatment of CFH. Considering the patient's history of congenital talipes equinovalgus, her condition aligns with Paley type IIIB. According to Paley's reconstructive methods, type III patients should first correct foot and ankle deformities to achieve ankle joint stability before performing tibial osteotomy and lengthening.

Paley's method involves using subtalar joint osteotomy and tibial osteotomy for the correction of ankle deformities in children. Other methods to achieve ankle joint stability include: I. H. Thomas (22) applied the Gruca procedure in children with distal fibular aplasia, performing reconstructive osteotomy of the ankle to maintain balance and prevent secondary valgus deformity during development. Wiltze (23) utilized osteotomy to correct ankle valgus deformity without causing abnormal medial malleolar prominence or limb shortening, recommending osteotomy be performed after skeletal maturity. Catagni et al. (24) reported 18 cases of complete fibular aplasia, with 2 cases ultimately undergoing ankle arthrodesis, showing favorable long-term follow-up outcomes. Additionally, Bishay et al. combined fibular band resection, soft tissue release, and Ilizarov technique to stabilize the ankle joint (25). In this case, the family found that the child's right foot was obviously valgus since birth. The parents applied homemade splints to immobilize the right ankle in a functional position for 6 consecutive months. After 6 months of treatment, the external appearance of the right foot normalized. The patient achieved independent ambulation at 18 months of age. As the patient grew up, the bilateral lower limb length discrepancy progressively worsened, prompting the current consultation. Although the fibula was completely absent, the patient presented with functional ambulation capacity (FAC 5) at the time of admission, with a 40 cm step length, a negative anterior drawer test, and a normal one-leg standing test. Therefore, the affected ankle joint was considered to be in a stable state and no ankle reconstruction surgery was needed. Therefore, we chose to achieve limb equilength by tibial osteotomy and lengthening using Ilizarov technique. Latest follow-up results indicate satisfactory lengthening of the right tibia in the patient.

## 4 Conclusions

Although classification systems and surgical techniques vary, the ultimate objective is to achieve stable ankle and knee joints, as well as well-aligned and equal-length lower limbs in patients. Normal ambulation is the ultimate goal of CFH treatment and management. To the best of our knowledge, this is the first reported case of CFH with complete absence of fibula in which ankle stability can be maintained after prior conservative treatment. This also suggests that early detection and early conservative treatment for ankle dysplasia may be able to reduce the pain associated with multiple surgeries experienced by the patients. However, as this is a single-case report with inherent limitations, outcomes may vary among CFH patients. The efficacy and feasibility of early conservative management require validation through multicenter, large-sample, prospective clinical randomized controlled trials. We believe this report not only emphasizes the importance of early screening and conservative management for CFH but also provides novel therapeutic insights for CFH treatment.

## Data Availability

The original contributions presented in the study are included in the article/Supplementary Material, further inquiries can be directed to the corresponding author.
